# Crystal structure of non-centrosymmetric bis­(4-meth­oxy­benzyl­ammonium) tetra­chlorido­zincate

**DOI:** 10.1107/S2056989016010069

**Published:** 2016-06-24

**Authors:** Najla Mahbouli Rhouma, Ali Rayes, Francesco Mezzadri, Gianluca Calestani, Mohamed Loukil

**Affiliations:** a Laboratoire des Sciences des Matériaux et d’Environnement, Faculté des Sciences, Université de Sfax, BP 1171, Route de Soukra, 3018 Sfax, Tunisia; b Unité de Recherche, Catalyse et Matériaux pour l’Environnement et les Procédés, URCMEP, (UR11ES85), Faculté des Sciences de Gabès, Campus Universitaire, 6072 Gabès, Tunisia; c Dipartimento di Chimica, Universitá di Parma, Parco Area delle Scienze 17A, I-43124 Parma, Italy

**Keywords:** crystal structure, non-centrosymmetric organic–inorganic hybrid material, hydrogen bonds

## Abstract

The structure of the title non-centrosymmetric organic–inorganic hybrid salt, (C_8_H_12_NO)_2_[ZnCl_4_], consists of two 4-meth­oxy­benzyl­ammonium cations sandwiched between anionic layers, formed by isolated tetra­chlorido­zincate tetra­hedra. The double layers extend parallel to the *ac* plane. The crystal packing is assured by Coulombic inter­actions and by a complex N—H⋯Cl and C—H⋯Cl hydrogen-bonding system mostly involving the positively charged ammonium groups and the chloride ligands of the isolated tetra­hedral [ZnCl_4_]^2−^ units. One of the methyl­ene­ammonium groups is disordered over two sets of sites in a 0.48 (2):0.52 (2) ratio. The crystal investigated was twinned by non-merohedry with a twin component ratio of 0.738 (2):0.262 (2).

## Abstract 2

The crystal structure of the new non-centrosymmetric organic–inorganic hybrid salt (C_8_H_12_NO)_2_[ZnCl_4_], consists of 4-meth­oxy­benzyl­ammonium cations sandwiched between tetra­chlorido­zincate anionic layers running parallel to the *ac* plane.

## Chemical context   

Non-linear optical (NLO) materials have received much attention in different research areas due to their potential applications in high-density optical data storage, electro-optical shutters, optical communication and signal processing (Maury & Le Bozec, 2005 ▸; Green *et al.*, 2011 ▸; Evans & Lin, 2002 ▸). Mostly connected in the past to a few families of inorganic materials, the research was then extended to organic materials, generally salts of amino acids with organic acids, which are expected to have relatively strong NLO properties due to delocalized electrons at π–π* orbitals. More recently, organic–inorganic hybrid materials showing non-centrosymmetric structures started gaining attention in the field, since they are expected to offer enhanced properties, such as second harmonic generation efficiency, by combining the characteristic features of both organic and inorganic moieties. These materials are usually constituted by the crystal packing of inorganic anions (typically halogenidometalates) and organic ammonium cations ensured by hydrogen bonds and Coulombic inter­actions (Brammer *et al.*, 2002 ▸). Herein we report the synthesis and crystal structure of a new organic–inorganic hybrid compound, bis­(4-meth­oxy­benzyl­ammonium) tetra­chlorido­zincate. This salt crystallizes in a non-centrosymmetric space group and hence could be a potential candidate for second order non-linear optical properties.
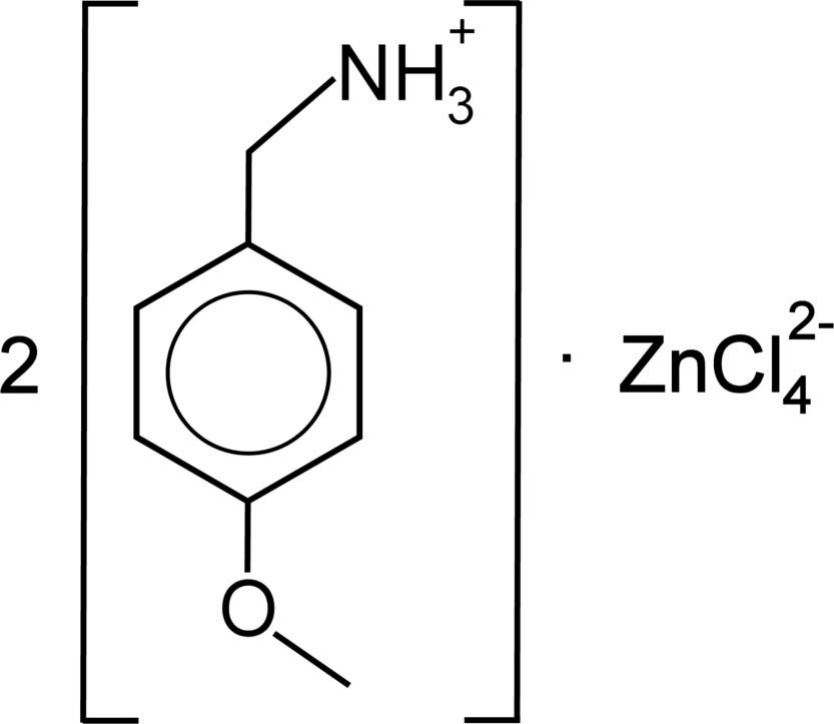


## Structural commentary   

**Figure 1:**
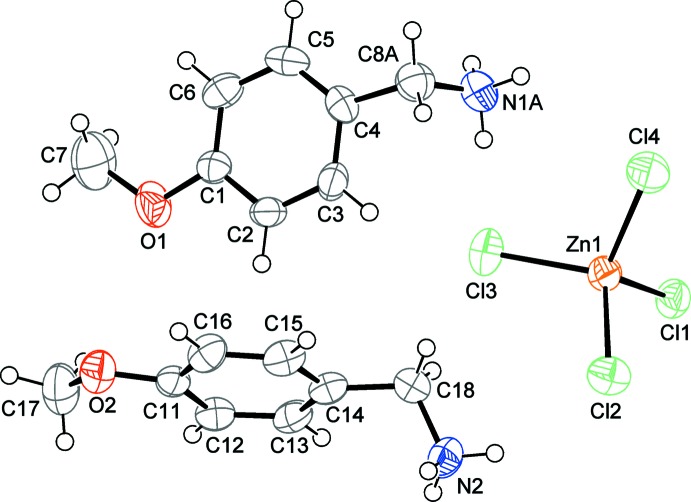
The asymmetric unit of the title compound with displacement ellipsoids drawn at the 50% probability level. Only the major component of the disordered methyl­ene­ammonium group is shown for clarity.

The asymmetric unit of the crystal structure consists of an isolated tetra­chlorido­zincate anion, [ZnCl_4_]^2−^ and two 4-meth­oxy­benzyl­ammonium cations, (C_8_H_12_NO)^+^, as shown in Fig. 1 ▸. One of the cations shows positional disorder of the methyl­ene­ammonium moiety. The lengths of the C—C, C—N and C—O bonds in the two independent 4-meth­oxy­benzyl­ammonium cations are in accordance with corresponding distances found in the literature (Groom *et al.*, 2016 ▸). The Zn^II^ atom is tetra­hedrally coordinated by four chloride ligands with Zn—Cl bond lengths ranging from 2.249 (2) to 2.289 (2) Å and Cl—Zn—Cl bond angles varying between 107.25 (8) and 112.41 (10)°.

## Supra­molecular features   

**Figure 2:**
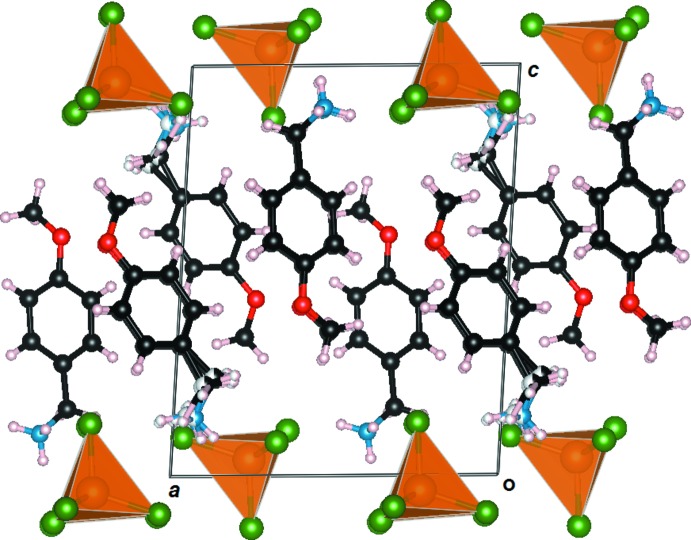
Packing diagram of the title compound viewed along the *b* axis, showing the alternate stacking, along the *c* axis, of organic and inorganic layers.

**Figure 3:**
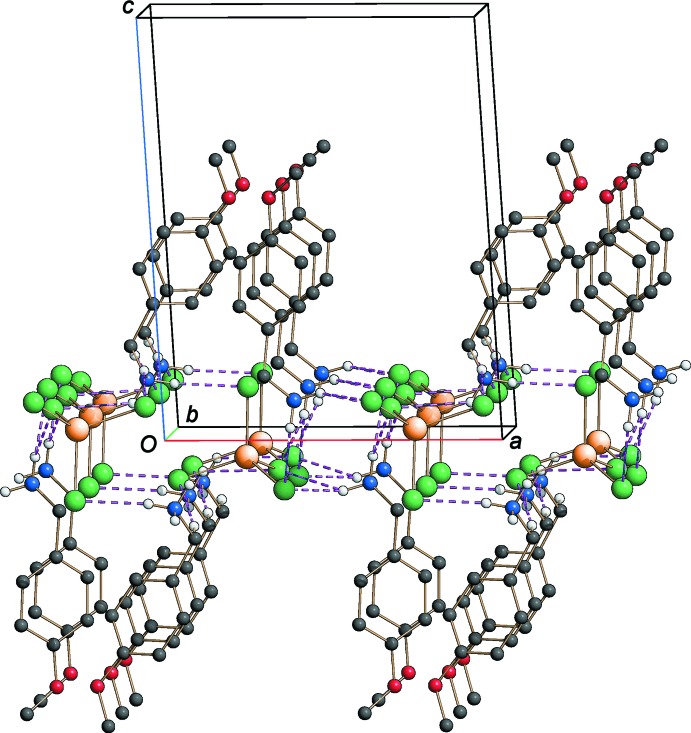
Partial packing diagram of the title compound approximately viewed along the *b* axis, showing the hydrogen-bonding network (dashed lines).

The crystal structure consist of 4-meth­oxy­benzyl­ammonium cations sandwiched between tetra­chlorido­zincate layers extending parallel to the *ac* plane, as shown in Fig. 2 ▸. The cationic units are linked into a two-dimensional network by weak C—H⋯π inter­actions (Table 1 ▸). The crystal packing is assured by a complex hydrogen-bonding system, mostly involving the positively charged ammonium groups and the chloride ligands of the isolated tetra­hedral [ZnCl_4_]^2−^ units (Table 1 ▸), which reinforce the Coulombic inter­actions, as depicted in Fig. 3 ▸. Whereas the N2 atom is blocked by a very efficient hydrogen-bonding system involving five donor⋯acceptor distances ranging from 3.279 (8) to 3.452 (7) Å, the N1 ammonium group is disordered over two sets of sites as a consequence of a less efficient hydrogen bonding.

## Database survey   

A search of the Cambridge Structural Database (Version 5.37; last update February 2016; Groom *et al.*, 2016 ▸) for related compounds showed the occurrence of the cadmium analogue of formula (C_8_H_12_NO)_2_[CdCl_4_] (Kefi *et al.*, 2011 ▸), in which the coordination sphere of the metal is octa­hedral, giving rise to the formation of perovskite-like edge-sharing units that built up two-dimensional anionic layers parallel to the *bc* plane.

## Synthesis and crystallization   

Single crystals of (C_8_H_12_NO)_2_[ZnCl_4_] were synthesized starting from 4-meth­oxy­benzyl­amine (Sigma–Aldrich, 98%), zinc chloride (Sigma–Aldrich, 98%) and HCl (37%), which were weighted in the stoichiometric proportion conforming to the equation reaction:

2 C_8_H_11_NO + 2 HCl + ZnCl_2_ → (C_8_H_12_NO)_2_[ZnCl_4_]

After mixing the reagents in 50 ml of water and stirring at room temperature for more 3 h, the resulting solution was placed in a Petri dish and allowed to evaporate slowly. Single crystals suitable for X-ray diffraction were obtained within a week (yield: 75%).

## Refinement   

Crystal data, data collection and structure refinement details are summarized in Table 2 ▸. The crystals of bis­(4-meth­oxy­benzyl­ammonium) tetra­chlorido­zincate were systematically affected by non-merohedral polar twinning. The ratio of the twin components of the crystal selected for X-ray analysis was refined to 0.738 (2):0.262 (2). One methyl­ene­ammonium group was found to be disordered over two sets of sites with a refined occupancy ratio of 0.52 (2):0.48 (2). During the refinement of the disordered group, the C—C and C—N bond lengths were constrained to be 1.50 (2) and 1.47 (1) Å, respectively. EADP and ISOR restraints (Sheldrick, 2015*b* ▸) were also applied. All H atoms were placed geometrically and refined using a riding-model approximation, with C—H = 0.93–0.97 Å, N—H = 0.89 Å, and with *U*_iso_(H) = 1.2*U*_eq_(C) or 1.5*U*_eq_(C, N) for methyl and ammonium H atoms, for which a rotating model was applied.

## Supplementary Material

Crystal structure: contains datablock(s) I. DOI: 10.1107/S2056989016010069/wm5302sup1.cif

Structure factors: contains datablock(s) I. DOI: 10.1107/S2056989016010069/wm5302Isup2.hkl

CCDC reference: 1486826

Additional supporting information: crystallographic information; 3D view; checkCIF report

## Figures and Tables

**Table 1:** Hydrogen-bond geometry (Å, °) *Cg*1 and *Cg*2 are the centroids of the C11–C16 and C1–C6 rings, respectively

*D*—H⋯*A*	*D*—H	H⋯*A*	*D*⋯*A*	*D*—H⋯*A*
N1*A*—H1*A*1⋯Cl3	0.89	2.32	3.19 (2)	164
N1*A*—H1*A*2⋯Cl2^i^	0.89	2.75	3.26 (2)	118
N1*A*—H1*A*3⋯Cl4^i^	0.89	2.64	3.34 (2)	137
C8*A*—H8*A*2⋯Cl4^ii^	0.97	2.77	3.72 (2)	168
N1*B*—H1*B*1⋯Cl4^ii^	0.89	2.78	3.61 (3)	154
N1*B*—H1*B*2⋯Cl2^i^	0.89	2.66	3.33 (2)	133
N1*B*—H1*B*3⋯Cl3	0.89	2.80	3.45 (2)	131
C8*B*—H8*B*2⋯Cl1^ii^	0.97	2.82	3.60 (2)	138
N2—H1*N*⋯Cl1^iii^	0.89	2.65	3.364 (7)	138
N2—H1*N*⋯Cl2^iii^	0.89	2.75	3.336 (7)	125
N2—H2*N*⋯Cl3^iv^	0.89	2.45	3.279 (8)	156
N2—H3*N*⋯Cl1^iv^	0.89	2.72	3.331 (7)	127
N2—H3*N*⋯Cl2	0.89	2.71	3.452 (7)	141
C2—H2⋯*Cg*1	0.93	2.62	3.432 (8)	146
C6—H6⋯*Cg*2^i^	0.93	2.86	3.579 (8)	135

Symmetry codes: (i) 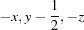; (ii) 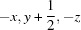; (iii) 
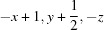
; (iv) 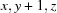.

**Table 2:** Experimental details

Crystal data	
Chemical formula	(C_8_H_12_NO)_2_[ZnCl_4_]
*M*_r_	483.54
Crystal system, space group	Monoclinic, *P*2_1_
Temperature (K)	294
*a*, *b*, *c* (Å)	10.6849 (10), 7.4540 (7), 13.3961 (12)
β (°)	93.482 (2)
*V* (Å^3^)	1064.97 (17)
*Z*	2
Radiation type	Mo *K*α
μ (mm^−1^)	1.67
Crystal size (mm)	0.31 × 0.29 × 0.11

Data collection	
Diffractometer	Bruker SMART CCD
Absorption correction	Multi-scan (*SADABS*; Bruker, 2008 ▸)
*T*_min_, *T*_max_	0.604, 0.827
No. of measured, independent and observed [*I* > 2σ(*I*)] reflections	2132, 2132, 1932
(sin θ/λ)_max_ (Å^−1^)	0.606

Refinement	
*R*[*F* ^2^ > 2σ(*F* ^2^)], *wR*(*F* ^2^), *S*	0.043, 0.108, 1.08
No. of reflections	2132
No. of parameters	239
No. of restraints	29
H-atom treatment	H-atom parameters constrained
Δρ_max_, Δρ_min_ (e Å^−3^)	0.42, −0.44
Absolute structure	No quotients, so Flack parameter determined by classical intensity fit
Absolute structure parameter	0.09 (2)

Computer programs: *APEX2* and *SAINT* (Bruker, 2008 ▸), *SHELXT* (Sheldrick, 2015*a* ▸), *SHELXL2014* (Sheldrick, 2015*b* ▸), *ORTEP-3 for Windows* (Farrugia, 2012 ▸), *VESTA* (Momma & Izumi, 2011 ▸) and *SCHAKAL* (Keller, 1999 ▸).

## supplementary crystallographic information

### Crystal data

**Table:**

(C_8_H_12_NO)_2_[ZnCl_4_]	*F*(000) = 496
*M**_r_* = 483.54	*D*_x_ = 1.508 Mg m^−^^3^
Monoclinic, *P*2_1_	Mo *K*α radiation, λ = 0.71073 Å
*a* = 10.6849 (10) Å	Cell parameters from 196 reflections
*b* = 7.4540 (7) Å	θ = 7.3–17.5°
*c* = 13.3961 (12) Å	µ = 1.67 mm^−^^1^
β = 93.482 (2)°	*T* = 294 K
*V* = 1064.97 (17) Å^3^	Prism, colourless
*Z* = 2	0.31 × 0.29 × 0.11 mm

### Data collection

**Table:**

Bruker SMART CCD diffractometer	1932 reflections with *I* > 2σ(*I*)
ω scan	θ_max_ = 25.5°, θ_min_ = 1.5°
Absorption correction: multi-scan (*SADABS*; Bruker, 2008)	*h* = −12→12
*T*_min_ = 0.604, *T*_max_ = 0.827	*k* = 0→9
2132 measured reflections	*l* = 0→16
2132 independent reflections	

### Refinement

**Table:**

Refinement on *F*^2^	Hydrogen site location: inferred from neighbouring sites
Least-squares matrix: full	H-atom parameters constrained
*R*[*F*^2^ > 2σ(*F*^2^)] = 0.043	*w* = 1/[σ^2^(*F*_o_^2^) + (0.0576*P*)^2^ + 0.2617*P*] where *P* = (*F*_o_^2^ + 2*F*_c_^2^)/3
*wR*(*F*^2^) = 0.108	(Δ/σ)_max_ < 0.001
*S* = 1.08	Δρ_max_ = 0.42 e Å^−^^3^
2132 reflections	Δρ_min_ = −0.44 e Å^−^^3^
239 parameters	Absolute structure: No quotients, so Flack parameter determined by classical intensity fit
29 restraints	Absolute structure parameter: 0.09 (2)

### Special details

**Table:**

Geometry. All esds (except the esd in the dihedral angle between two l.s. planes) are estimated using the full covariance matrix. The cell esds are taken into account individually in the estimation of esds in distances, angles and torsion angles; correlations between esds in cell parameters are only used when they are defined by crystal symmetry. An approximate (isotropic) treatment of cell esds is used for estimating esds involving l.s. planes.
Refinement. Refined as a 2-component twin.

### Fractional atomic coordinates and isotropic or equivalent isotropic displacement parameters (Å^2^)

**Table:**

	*x*	*y*	*z*	*U*_iso_*/*U*_eq_	Occ. (<1)
Zn1	0.23731 (7)	0.06114 (12)	−0.04066 (6)	0.0413 (2)	
Cl1	0.35118 (19)	−0.1525 (3)	−0.11156 (13)	0.0464 (5)	
Cl2	0.3173 (2)	0.3349 (3)	−0.08077 (16)	0.0515 (5)	
Cl3	0.2564 (2)	0.0169 (3)	0.12644 (14)	0.0541 (5)	
Cl4	0.03137 (18)	0.0515 (5)	−0.09460 (17)	0.0714 (7)	
O1	0.2318 (5)	0.1370 (9)	0.5695 (4)	0.0585 (16)	
O2	0.3732 (5)	0.6144 (8)	0.5857 (4)	0.0541 (15)	
N1A	−0.039 (2)	−0.010 (3)	0.1458 (15)	0.060 (4)	0.52 (2)
H1A1	0.0398	0.0184	0.1345	0.090*	0.52 (2)
H1A2	−0.0863	−0.0023	0.0889	0.090*	0.52 (2)
H1A3	−0.0418	−0.1214	0.1693	0.090*	0.52 (2)
C8A	−0.087 (2)	0.115 (3)	0.2197 (13)	0.051 (4)	0.52 (2)
H8A1	−0.1741	0.0889	0.2288	0.062*	0.52 (2)
H8A2	−0.0816	0.2370	0.1945	0.062*	0.52 (2)
N1B	−0.062 (2)	0.077 (4)	0.1398 (13)	0.060 (4)	0.48 (2)
H1B1	−0.0269	0.1835	0.1303	0.090*	0.48 (2)
H1B2	−0.1227	0.0581	0.0926	0.090*	0.48 (2)
H1B3	−0.0039	−0.0084	0.1365	0.090*	0.48 (2)
C8B	−0.1142 (17)	0.073 (4)	0.2386 (12)	0.051 (4)	0.48 (2)
H8B1	−0.1541	−0.0422	0.2483	0.062*	0.48 (2)
H8B2	−0.1775	0.1656	0.2421	0.062*	0.48 (2)
N2	0.4066 (6)	0.6363 (10)	0.1058 (5)	0.0491 (16)	
H1N	0.4869	0.6150	0.1236	0.074*	
H2N	0.3897	0.7516	0.1158	0.074*	
H3N	0.3916	0.6099	0.0414	0.074*	
C1	0.1468 (6)	0.1175 (9)	0.4901 (5)	0.0369 (16)	
C2	0.1783 (7)	0.2035 (10)	0.4051 (5)	0.0424 (18)	
H2	0.2535	0.2663	0.4048	0.051*	
C3	0.0987 (8)	0.1978 (11)	0.3188 (6)	0.0456 (18)	
H3	0.1199	0.2572	0.2612	0.055*	
C4	−0.0124 (7)	0.1024 (10)	0.3201 (5)	0.0409 (18)	
C5	−0.0412 (7)	0.0166 (11)	0.4056 (6)	0.049 (2)	
H5	−0.1157	−0.0477	0.4061	0.059*	
C6	0.0374 (7)	0.0223 (10)	0.4917 (6)	0.047 (2)	
H6	0.0162	−0.0372	0.5493	0.057*	
C7	0.2037 (11)	0.0551 (19)	0.6615 (7)	0.085 (3)	
H7A	0.1208	0.0892	0.6781	0.128*	
H7B	0.2634	0.0939	0.7135	0.128*	
H7C	0.2078	−0.0729	0.6549	0.128*	
C11	0.3734 (7)	0.5951 (10)	0.4849 (5)	0.0393 (16)	
C12	0.4613 (7)	0.4999 (11)	0.4364 (6)	0.0423 (18)	
H12	0.5296	0.4487	0.4721	0.051*	
C13	0.4466 (7)	0.4808 (12)	0.3325 (6)	0.0450 (19)	
H13	0.5050	0.4138	0.2997	0.054*	
C14	0.3470 (6)	0.5594 (13)	0.2768 (5)	0.0440 (16)	
C15	0.2628 (7)	0.6580 (10)	0.3274 (6)	0.0472 (18)	
H15	0.1967	0.7140	0.2912	0.057*	
C16	0.2727 (8)	0.6769 (10)	0.4303 (6)	0.0468 (18)	
H16	0.2134	0.7427	0.4629	0.056*	
C17	0.4625 (10)	0.5122 (14)	0.6466 (6)	0.070 (3)	
H17A	0.4541	0.3873	0.6299	0.105*	
H17B	0.4473	0.5292	0.7158	0.105*	
H17C	0.5458	0.5517	0.6346	0.105*	
C18	0.3266 (7)	0.5243 (12)	0.1665 (6)	0.054 (2)	
H18A	0.3437	0.3989	0.1535	0.065*	
H18B	0.2394	0.5470	0.1461	0.065*	

### Atomic displacement parameters (Å^2^)

**Table:**

	*U*^11^	*U*^22^	*U*^33^	*U*^12^	*U*^13^	*U*^23^
Zn1	0.0397 (4)	0.0455 (4)	0.0384 (4)	−0.0002 (5)	0.0005 (4)	0.0012 (4)
Cl1	0.0524 (11)	0.0456 (9)	0.0416 (9)	0.0014 (9)	0.0070 (9)	−0.0042 (9)
Cl2	0.0516 (11)	0.0415 (10)	0.0607 (11)	0.0008 (10)	−0.0015 (10)	0.0091 (9)
Cl3	0.0695 (12)	0.0560 (13)	0.0369 (9)	0.0119 (11)	0.0042 (10)	0.0018 (8)
Cl4	0.0364 (9)	0.115 (2)	0.0623 (12)	−0.0014 (15)	−0.0031 (9)	−0.0049 (16)
O1	0.056 (3)	0.076 (4)	0.042 (3)	0.006 (3)	−0.014 (3)	0.001 (3)
O2	0.063 (3)	0.056 (4)	0.044 (3)	0.002 (3)	0.007 (3)	−0.004 (3)
N1A	0.056 (7)	0.077 (12)	0.047 (4)	−0.011 (9)	−0.007 (5)	−0.003 (8)
C8A	0.047 (6)	0.055 (7)	0.052 (6)	0.001 (5)	−0.002 (5)	0.004 (5)
N1B	0.056 (7)	0.077 (12)	0.047 (4)	−0.011 (9)	−0.007 (5)	−0.003 (8)
C8B	0.047 (6)	0.055 (7)	0.052 (6)	0.001 (5)	−0.002 (5)	0.004 (5)
N2	0.051 (4)	0.058 (4)	0.038 (3)	0.006 (3)	0.006 (3)	0.007 (3)
C1	0.033 (4)	0.037 (4)	0.041 (4)	0.003 (3)	0.004 (3)	−0.001 (3)
C2	0.037 (4)	0.043 (4)	0.048 (4)	−0.010 (3)	0.010 (3)	−0.006 (3)
C3	0.058 (5)	0.048 (4)	0.031 (4)	−0.005 (4)	0.008 (3)	−0.001 (3)
C4	0.041 (4)	0.040 (5)	0.041 (4)	0.003 (3)	−0.004 (3)	−0.008 (3)
C5	0.034 (4)	0.044 (5)	0.069 (5)	−0.010 (3)	0.005 (4)	−0.002 (4)
C6	0.052 (5)	0.038 (4)	0.053 (4)	0.000 (4)	0.014 (4)	0.010 (3)
C7	0.105 (8)	0.086 (7)	0.062 (5)	0.012 (9)	−0.016 (6)	0.013 (7)
C11	0.044 (4)	0.034 (4)	0.041 (4)	−0.010 (4)	0.007 (3)	−0.002 (3)
C12	0.036 (4)	0.038 (4)	0.052 (5)	0.002 (3)	0.004 (4)	0.005 (4)
C13	0.038 (4)	0.047 (4)	0.052 (5)	0.000 (4)	0.016 (4)	0.000 (4)
C14	0.043 (4)	0.042 (4)	0.048 (4)	−0.006 (5)	0.010 (3)	0.007 (4)
C15	0.042 (4)	0.043 (4)	0.057 (4)	0.004 (4)	0.007 (4)	0.010 (4)
C16	0.051 (4)	0.039 (4)	0.052 (4)	0.006 (4)	0.015 (4)	−0.002 (3)
C17	0.093 (7)	0.071 (7)	0.045 (5)	0.000 (6)	−0.004 (5)	0.001 (5)
C18	0.048 (4)	0.067 (6)	0.047 (4)	−0.016 (4)	0.002 (4)	−0.006 (4)

### Geometric parameters (Å, º)

**Table:**

Zn1—Cl1	2.249 (2)	C2—C3	1.393 (11)
Zn1—Cl3	2.2595 (19)	C2—H2	0.9300
Zn1—Cl4	2.275 (2)	C3—C4	1.385 (11)
Zn1—Cl2	2.289 (2)	C3—H3	0.9300
O1—C1	1.363 (8)	C4—C5	1.363 (11)
O1—C7	1.424 (11)	C5—C6	1.385 (11)
O2—C11	1.359 (9)	C5—H5	0.9300
O2—C17	1.435 (11)	C6—H6	0.9300
N1A—C8A	1.473 (10)	C7—H7A	0.9600
N1A—H1A1	0.8900	C7—H7B	0.9600
N1A—H1A2	0.8900	C7—H7C	0.9600
N1A—H1A3	0.8900	C11—C12	1.371 (11)
C8A—C4	1.523 (15)	C11—C16	1.403 (11)
C8A—H8A1	0.9700	C12—C13	1.398 (11)
C8A—H8A2	0.9700	C12—H12	0.9300
N1B—C8B	1.469 (10)	C13—C14	1.392 (11)
N1B—H1B1	0.8900	C13—H13	0.9300
N1B—H1B2	0.8900	C14—C15	1.372 (11)
N1B—H1B3	0.8900	C14—C18	1.504 (10)
C8B—C4	1.510 (15)	C15—C16	1.384 (11)
C8B—H8B1	0.9700	C15—H15	0.9300
C8B—H8B2	0.9700	C16—H16	0.9300
N2—C18	1.474 (10)	C17—H17A	0.9600
N2—H1N	0.8900	C17—H17B	0.9600
N2—H2N	0.8900	C17—H17C	0.9600
N2—H3N	0.8900	C18—H18A	0.9700
C1—C2	1.366 (10)	C18—H18B	0.9700
C1—C6	1.369 (10)		
			
Cl1—Zn1—Cl3	107.25 (8)	C5—C4—C3	119.2 (7)
Cl1—Zn1—Cl4	112.41 (10)	C5—C4—C8B	110.5 (11)
Cl3—Zn1—Cl4	109.72 (9)	C3—C4—C8B	130.3 (11)
Cl1—Zn1—Cl2	108.22 (8)	C5—C4—C8A	129.8 (11)
Cl3—Zn1—Cl2	110.50 (9)	C3—C4—C8A	110.9 (11)
Cl4—Zn1—Cl2	108.73 (11)	C4—C5—C6	122.0 (7)
C1—O1—C7	117.6 (7)	C4—C5—H5	119.0
C11—O2—C17	117.9 (6)	C6—C5—H5	119.0
C8A—N1A—H1A1	109.5	C1—C6—C5	118.5 (7)
C8A—N1A—H1A2	109.5	C1—C6—H6	120.7
H1A1—N1A—H1A2	109.5	C5—C6—H6	120.7
C8A—N1A—H1A3	109.5	O1—C7—H7A	109.5
H1A1—N1A—H1A3	109.5	O1—C7—H7B	109.5
H1A2—N1A—H1A3	109.5	H7A—C7—H7B	109.5
N1A—C8A—C4	111.8 (14)	O1—C7—H7C	109.5
N1A—C8A—H8A1	109.3	H7A—C7—H7C	109.5
C4—C8A—H8A1	109.3	H7B—C7—H7C	109.5
N1A—C8A—H8A2	109.3	O2—C11—C12	124.6 (7)
C4—C8A—H8A2	109.3	O2—C11—C16	115.1 (6)
H8A1—C8A—H8A2	107.9	C12—C11—C16	120.3 (6)
C8B—N1B—H1B1	109.5	C11—C12—C13	119.0 (7)
C8B—N1B—H1B2	109.5	C11—C12—H12	120.5
H1B1—N1B—H1B2	109.5	C13—C12—H12	120.5
C8B—N1B—H1B3	109.5	C14—C13—C12	121.7 (7)
H1B1—N1B—H1B3	109.5	C14—C13—H13	119.1
H1B2—N1B—H1B3	109.5	C12—C13—H13	119.1
N1B—C8B—C4	110.6 (15)	C15—C14—C13	117.7 (7)
N1B—C8B—H8B1	109.5	C15—C14—C18	121.2 (7)
C4—C8B—H8B1	109.5	C13—C14—C18	120.9 (7)
N1B—C8B—H8B2	109.5	C14—C15—C16	122.3 (8)
C4—C8B—H8B2	109.5	C14—C15—H15	118.9
H8B1—C8B—H8B2	108.1	C16—C15—H15	118.9
C18—N2—H1N	109.5	C15—C16—C11	119.0 (7)
C18—N2—H2N	109.5	C15—C16—H16	120.5
H1N—N2—H2N	109.5	C11—C16—H16	120.5
C18—N2—H3N	109.5	O2—C17—H17A	109.5
H1N—N2—H3N	109.5	O2—C17—H17B	109.5
H2N—N2—H3N	109.5	H17A—C17—H17B	109.5
O1—C1—C2	114.5 (6)	O2—C17—H17C	109.5
O1—C1—C6	124.9 (7)	H17A—C17—H17C	109.5
C2—C1—C6	120.6 (7)	H17B—C17—H17C	109.5
C1—C2—C3	120.6 (7)	N2—C18—C14	112.9 (7)
C1—C2—H2	119.7	N2—C18—H18A	109.0
C3—C2—H2	119.7	C14—C18—H18A	109.0
C4—C3—C2	119.1 (7)	N2—C18—H18B	109.0
C4—C3—H3	120.5	C14—C18—H18B	109.0
C2—C3—H3	120.5	H18A—C18—H18B	107.8

### Hydrogen-bond geometry (Å, º)

*Cg*1 and *Cg*2 are the centroids of the C11–C16 and C1–C6 rings, respectively

**Table:**

*D*—H···*A*	*D*—H	H···*A*	*D*···*A*	*D*—H···*A*
N1*A*—H1*A*1···Cl3	0.89	2.32	3.19 (2)	164
N1*A*—H1*A*2···Cl2^i^	0.89	2.75	3.26 (2)	118
N1*A*—H1*A*3···Cl4^i^	0.89	2.64	3.34 (2)	137
C8*A*—H8*A*2···Cl4^ii^	0.97	2.77	3.72 (2)	168
N1*B*—H1*B*1···Cl4^ii^	0.89	2.78	3.61 (3)	154
N1*B*—H1*B*2···Cl2^i^	0.89	2.66	3.33 (2)	133
N1*B*—H1*B*3···Cl3	0.89	2.80	3.45 (2)	131
C8*B*—H8*B*2···Cl1^ii^	0.97	2.82	3.60 (2)	138
N2—H1*N*···Cl1^iii^	0.89	2.65	3.364 (7)	138
N2—H1*N*···Cl2^iii^	0.89	2.75	3.336 (7)	125
N2—H2*N*···Cl3^iv^	0.89	2.45	3.279 (8)	156
N2—H3*N*···Cl1^iv^	0.89	2.72	3.331 (7)	127
N2—H3*N*···Cl2	0.89	2.71	3.452 (7)	141
C2—H2···*Cg*1	0.93	2.62	3.432 (8)	146
C6—H6···*Cg*2^i^	0.93	2.86	3.579 (8)	135

Symmetry codes: (i) −*x*, *y*−1/2, −*z*; (ii) −*x*, *y*+1/2, −*z*; (iii) −*x*+1, *y*+1/2, −*z*; (iv) *x*, *y*+1, *z*.

## References

[bb1] Brammer, L., Swearingen, J. K., Bruton, E. Z. & Sherwood, P. (2002). *Proc. Nat. Acad. Sci. USA*, **99**, 4956–4961. 10.1073/pnas.072623399 PMC122702 11959946

[bb2] Bruker (2008). *APEX2*, *SAINT* and *SADABS*. Bruker AXS Inc., Madison, Wisconsin, USA.

[bb3] Evans, O. R. & Lin, W. B. (2002). *Acc. Chem. Res.* **35**, 511–522. 10.1021/ar0001012 12118990

[bb4] Farrugia, L. J. (2012). *J. Appl. Cryst.* **45**, 849–854.

[bb5] Green, K. A., Cifuentes, M. P., Samoc, M. & Humphrey, M. G. (2011). *Coord. Chem. Rev.* **255**, 2530–2541.

[bb6] Groom, C. R., Bruno, I. J., Lightfoot, M. P. & Ward, S. C. (2016). *Acta Cryst.* B**72**, 171–179. 10.1107/S2052520616003954 PMC4822653 27048719

[bb7] Kefi, R., Maher, E. G., Zeller, M., Lefebvre, F. & Ben Nasr, C. (2011). Private communication (refcode XASKEJ). CCDC, Cambridge, England.

[bb8] Keller, E. (1999). *SCHAKAL*. University of Freiburg, Germany.

[bb9] Maury, O. & Le Bozec, H. (2005). *Acc. Chem. Res.* **38**, 691–704. 10.1021/ar020264l 16171312

[bb10] Momma, K. & Izumi, F. (2011). *J. Appl. Cryst.* **44**, 1272–1276.

[bb11] Sheldrick, G. M. (2015*a*). *Acta Cryst.* A**71**, 3–8.

[bb12] Sheldrick, G. M. (2015*b*). *Acta Cryst.* C**71**, 3–8.

